# Challenges in Accessing Health Care for People with Disability in the South Asian Context: A Review

**DOI:** 10.3390/ijerph15112366

**Published:** 2018-10-26

**Authors:** Venkata S. Murthy Gudlavalleti

**Affiliations:** 1 Indian Institute of Public Health & South Asia Centre for Disability Inclusive Development & Research, Hyderabad 500033, India; murthy.gvs@iiphh.org; Tel.: +91-40-49006001; 2 Public Health Eye Care & Disability, London School of Hygiene & Tropical Medicine, London WC1E7HT, UK; 3 Indian Institute of Public Health, ANV Arcade, 1 Amar Cooperative Society, Kavuri Hills, Madhapur, Hyderabad 500033, India

**Keywords:** access, barriers, disability, health care, India, South Asia

## Abstract

South Asia is a unique geopolitical region covering 3.4% of the world’s surface area and supporting 25% of the world’s population (1.75 billion). Available evidence from South Asia shows variable estimates of the magnitude of disability. The projected magnitude depends on whether an impairment focus is highlighted (approximately 1.6–2.1%) or functionality is given precedence (3.6–15.6%). People with disability (PWD) face significant challenges to accessing health care in the region. Studies show that adults with disability reported a four times higher incidence of a serious health problem in a year’s recall period. Evidence shows a significantly higher rate (17.8%) of hospitalization among PWD compared to others (5%). Chronic conditions like diabetes were also significantly higher. Women with disability had significantly more concerns on reproductive health issues. Studies from the South Asia region reveal that not only did PWD have a higher load of adverse health outcomes but they also faced significantly more barriers in accessing health services.

## 1. Introduction

Evidence on the magnitude of disability is crucial for effective planning and implementation of targeted interventions, and for dismantling barriers to mainstreaming people with disability (PWD) and improving their quality of life. The World Report on Disability highlights the need for data for developing strategies for PWD [1]. What is required is not just any data, but data using standardized definitions, because available data on disability varies widely due to lack of uniformity in defining disability, the inadequacy in scientific rigor in collecting the information, and the lack of adequately-powered sample sizes in estimating disability. Available data shows a wide variation with self-reporting during censuses showing figures of 1–2% while the World Report on Disability reports a global prevalence of 15% for disability [1]. 

The medical literature is replete with impairment-focused data. This does not consider an individual’s functionality that is required for day-to-day living. The visual acuity of two individuals may be the same, but, for example, the visual needs of an illiterate farmer differ significantly from a computer analyst. The Report categorically stated that impairment data are not an adequate proxy for disability and that measures need to be developed to obtain more comprehensive information on disability [1]. For planning effective programs at the district or local level, information is needed both on the impairments that need to be medically managed, and on functionality, integration, and stigma to develop community-specific interventions to mitigate the negative influences that reduce opportunities and access for PWD. 

## 2. Defining Disability

In the past disability was viewed solely as a ‘medical problem’ that needed to be ‘fixed’ appropriately by medicines, surgery, or rehabilitation. The role of society in creating a disabling attitudinal or physical environment that hindered people with disabilities to have equal opportunities was not appreciated [2]. This prompted the search for a valid universally acceptable definition that had flexibility to allow different uses and recognize the impact of the environment [3]. The International Classification of Functioning, Disability and Health (ICF) provided the framework to measure the relationship between the underlying health condition (disorder/disease) and its impact on body functions/structure, activity limitation, and social participation that can be influenced by environmental or personal factors (contextual factors) [4]. The World Report on Disability used this definition as the template to generate estimates on PWD [1]. The United Nations statistical division constituted a working group (called the Washington Group) to draft a universally acceptable definition of disability and its measurement [5]. They developed a set of questions called the Washington Group (WG) questionnaire to quantify the ICF concepts. The WG questionnaire is in use regularly, over the past decade to generate evidence on magnitude of PWD. This template helped develop other instruments like Rapid Assessment of Disability (RAD) [6], and the 34-item disability-screening questionnaire (DSQ-34) recently [7]. 

This review predominantly used the ICF definition of disability (activity limitation/social participation) wherever such data was available. Other sources of data are used if ICF targeted data was not available.

## 3. Health Concerns of People with Disability

There is ample evidence on the mitigating health circumstances that PWD face. Globally, irrespective of the economic development of a country, a significant proportion of the billion PWD have poorer health outcomes than those without disability [1]. Studies from the high as well as the low- and middle-income settings strongly endorse this observation [8,9,10,11,12,13,14,15]. The WHO Report also emphasizes that part of the problem with the poorer health outcomes also rests with the barriers to access to health care services, which discriminate against PWD [1]. Across all continents from the Americas to Australia, studies have documented barriers in accessing health care among PWD compared to those without disabilities, across the life spectrum [8,13,14,16,17,18,19,20,21,22,23,24,25,26]. Though the socio-economic milieu may be different, PWD have faced significantly more barriers than those without a disability in all contexts. Women with disability in Canada described multiple factors impeding access to health care [27]. PWD perceive that health providers and policy makers have preconceived notions about PWD’s capabilities, intentions, needs, and values. This they believe results in reduced health care access, as well as quality of health care [28]. Studies also show that women with disabilities report lower receipt of family planning services [22]. Evidence also documents that among PWD, the differentials in access is adverse for women, the poor, and those lacking health insurance cover [21].

## 4. Why Is South Asia of Interest?

South Asia is a unique geopolitical region which hosts a quarter of the world’s population and is home to the second (India), sixth (Pakistan), and the eighth (Bangladesh) most populous countries in the world and the largest number of poor people globally (500 million). Of a population of 1.75 billion residing in this region (2015), using the World Health Report estimate of 15% disability, there would be 270 million PWD though available statistics in the UNESCAP report shows a magnitude of 46 million, which could be an underestimation due to the data collection modalities adopted [28]. The region faces one of the world’s worst socio-economic inequities and there is poor coverage of basic health interventions with a significant difference between the highest and lowest socio-economic quintiles [29]. In a context where access to healthcare is affected by place of residence (urban/rural), gender, and socio-economic status for the general population, people with disability would find it significantly more challenging to access health care. South Asia harbors a significant proportion of global visual impairment [30], and hearing impairment [31]. It is interesting to note that a cohort study over a twenty-year period reported that Indian Asian PWD were significantly more likely to have poorer health outcomes compared to Europeans in the UK [32]. 

The review looks at the available evidence on magnitude of disability in the South Asia region and health outcomes and barriers to accessing health care in the region. 

## 5. Scope of the Review

In conducting the review, the following were included:

1. Literature published since 1998 AD (20 years reference period) from South Asia and other low- and middle-income countries.
2. Data from population-based studies so that a comparable denominator was available.
3. Studies reporting on all age populations or adult (18+ years) populations.
4. Different types of study instruments were included in the review. This included health surveys, targeted disability surveys, tools like the Rapid Assessment of Disability (RAD) tool, Census estimates and Washington Group (WG) criteria.

Studies including specific population segments (only children; those aged 50+ only; specific occupational categories etc.) or specific impairments were excluded. 

## 6. Magnitude of Disability in South Asia Region

As in the rest of the world, estimates of the prevalence and magnitude of disability in the South Asia Region are highly variable due to the lack of standardization of definitions of disability. Estimated prevalence rates of disability in the region range from a low of 1.9% (Census—all age), [33] to a high of 12.2% (Survey combining clinical examination with Washington Group (WG) criteria among 18+ years population [11]. Most of the studies in South Asia (Table 1) were from India. There was at least one estimate from all the countries in the South Asia region. 

Except in one study from India, [38] in all other studies, tools which measured functional status, (Rapid Assessment of Disability—RAD; Washington Group—WG) reported a higher prevalence than those which recorded self-reported impairments as a proxy for disability in census (Table 1). However in estimates collated from Sri Lanka, a wide variation was observed using the same tool (WG) at the same time period (2014–2015) [33,41]. This difference persisted despite the age cut-off adopted (all age versus 18+ years). Therefore, standardization of tools accompanied by adequate training to administer the tool and adequate quality assurance checks help generate valid data. The estimates from South Asia were comparable with reported prevalence rates from other LMICs (low- and middle-income countries) across the globe [11,28,42,43,44]. The available evidence therefore points to the situation being similar in most LMICs, but translating the prevalence rates into numbers results in South Asia harboring the largest pool of PWD in the world.

## 7. Health Status of People with Disability

Data on the health status, risk of disease and health outcomes of people with disabilities were compared with those without a disability (Table 2). Available evidence clearly indicates that the health needs of PWD were significantly more than those without a reported disability in India [14,16,36,37,38,45]. Respondents with disability also reported significantly more unmet needs compared to those without a disability [36,37,38]. In Afghanistan, it was observed that PWD visited health centers more often than those without a disability and the out-of-pocket expenses for PWD were higher [46]. A study from Bangladesh observed that PWD were 14 times more likely than others to seek treatment [47]. 

In the South Asia region, studies from India showed that the prevalence of non-communicable diseases (NCDs) such as diabetes and hypertension were significantly higher among PWD [14,15]. Similar findings were reported from other parts of Asia (Korea) too [10]. Physical impairments constitute a high proportion of PWD and with a sedentary lifestyle; risk of NCDs among these population subgroups will be high. This high risk of NCDs escaped attention earlier but with an increasing emphasis on these diseases and the flagging of the control of NCDs by the United Nations as part of the Sustainable Development Goals (SDGs), it is important to target PWD as a high-risk group for NCDs and SDGs in the future. This realization needs to be supported with uninterrupted medical supplies to ensure that the health of PWD is promoted.

A poorer health status of PWD was also observed in other LMICs too [8,23,26]. In South Africa, people with disability had a higher rate of unmet health needs as compared to non-disabled [19]. In Sierra Leone, persons with disabilities were more likely to use medication found in street markets (*p* < 0.011) and to try religious cures/prayers (*p* < 0.0001) as part of their medical treatment compared to those without a disability [26].

## 8. Specific Health Needs of Women with Disabilities

Among people with disabilities, women with disability are a special group as they have additional sexual and reproductive health needs and prenatal, natal, and post-natal care needs compared to other segments of the population. A study from India showed that a significantly lower proportion of women with disability experienced pregnancy (36.8%) compared to women without a disability (X^2^ –16.02; *p* < 0.001) [15]. The study also observed that there were no statistically significant differences between women with and without a disability with regard to utilization of antenatal care and pregnancy outcomes [15]. Similar observations have been reported from high-income countries also. 

## 9. Barriers to Accessing Health Care

Three major domains govern access to health care for PWD:

1. Individual characteristics including socio-economic factors and type and severity of impairments.
2. Nonmedical systemic factors including architectural designs, infrastructure, and affirmative action initiatives.
3. Provider perspectives and appreciation of the needs of PWD among the providers.

### 9.1. Individual Characteristics

Distance to a health facility, costs of care, transportation facilities, and lack of awareness about availability of services were flagged as major barriers to accessing health care in South Asia (Table 3) [14,35,36,37]. Cost of care and distance were the most significant individual level barriers reported across populations in other LMICs (low- and middle-income countries) [13,17,19,23].

### 9.2. Nonmedical Systemic Factors

There is limited evidence on the non-medical systemic factors from South Asia [14]. The limited available evidence showed that inadequate equipment/hospital infrastructure were of concern to PWD in India. In Pakistan, PWD with physical impairments reported significant physical barriers, due to the built environments, in accessing health services [48]. These included transportation, and outdoor and indoor environments in which health services are delivered, including buildings, waiting areas, washrooms, examination tables, beds etc. [48]. Transportation, and attitude of family members and the community were the main environmental barriers reported in Nepal [49].

PWD have poor access to preventive health services, which are a good measure of equity. In Pakistan, PWD had poor access to reproductive health care services and insufficient knowledge of preventive measures for tuberculosis, hepatitis, and HIV/AIDS [48].

### 9.3. Provider Perspectives of People with Disability

Available literature on the provider perspectives regarding PWD from South Asia has demonstrated a general lack of appreciation of the needs of PWD by providers and administrators. Ill-treatment by providers and the negative perceptions of PWD were important barriers to accessing services in South Asia [14,37]. In Nepal, providers’ attitudes towards disability were found to be negative with poor knowledge and skills about providing services to PWD [50].

## 10. Financial Barriers and Cost of Care

The inequities in access for PWD in many low- and middle-income countries gets camouflaged by the inadequate attention to health in general for the entire community with low allocations for health care in these countries. For example, in Afghanistan, the overall rate of health care utilization is low (25%) and this reduces the inequity differentials among population segments [51]. In India, catastrophic health expenditures increased over the period 1994–2014 across all sections of society but the households with an elderly person had a 3.6 times higher risk [52]. Disability is age-related and tends to be concentrated at older ages. Corroborating this point, another study from India observed that out-of-pocket expenditures were higher among households with a disabled elderly member [53]. The household income and expenditure survey data from Sri Lanka observed that multidimensional poverty among the households with disabled persons was higher than among other households [54]. Marginalized and disadvantaged groups in many LMICs face difficulties in accessing health services and this affects many sections of the population including PWD [23].

PWD are mostly dependent on their families for support including health care. In Pakistan 62% of men with disability and 87% of women with disability were financially dependent on their families and relatives [48]. In Nepal lack of funds for health expenses and the low socio-economic status of families of PWD were flagged as major financial barriers [49]. There is evidence to this effect from Nepal [55].

All countries in the South Asia region are LMICs. Population access to health care in general in these countries is sub-optimal and those with and without a disability are both disadvantaged. In such a milieu, all segments of the population have lower health expectations, as was demonstrated from a study in India [56].

The cost of health care is also a major concern for people with disability. A recent study from Bangladesh, analyzing data from the Bangladesh Household Income and Expenditure Survey, observed that out-of-pocket payments were significantly higher among individuals who reported a disability [57]. This data emphasizes the need for targeted financial protection for persons with disability, especially for the poorer populations. Similar observations were reported from Afghanistan where out-of-pocket expenditures were significantly higher for PWD [46].

## 11. Discussion 

The review documents that people with disabilities in South Asia have a high risk of suffering health problems, especially NCDs like diabetes, hypertension and ‘feeling low’ (as a proxy indicator for depression) etc. People with disabilities in South Asia have the same general health needs as others and they too need the same care for disease conditions like diarrhea, respiratory infections, viral fevers, malaria etc. However, unlike those without a disability, people with disability have additional health care needs. They need assistive devices/management for their underlying impairments, like polio, cleft palate, intellectual impairments, learning disabilities etc. They have a heightened risk of co-morbid conditions, especially non-communicable diseases and have more need of a counselling interface compared to those without a disability. Studies from South Asia and other LMICs show that the burden of poor health is accompanied by longer hospital stay [10,18] repeated hospitalization [9,13,14], and need for medication [14]. In Bangladesh, 85% PWD with physical impairments reported suffering from a general illness in a six-month recall period [58].

The travesty is that though people with disability have a higher risk of adverse health outcomes, their access to health services is hindered due to reasons beyond their control. This plays out at all levels of health care from the primary to the tertiary level. 

The Convention on Rights for Persons with Disability (CRPD) obligated states to provide equal access to health care for people with disabilities [59]. Article 25 of the declaration is devoted to health and states that health is a right for equal access to the highest attainable standard of health for people with disabilities and that governments should provide health services adapted to the needs of people with disability [58]. Sustainable Development Goals (SDG) also recognize that the inclusion of people with disabilities is critical for sustainable development [60]. The SDGs stress the need for improving access to healthcare services for all through Universal Health Coverage (UHC) [61]. This includes all the population sub segments including people with disability. As has been stated by some experts, if people with disabilities are not reached by health care initiatives it reflects on the fact that these efforts are ineffective [62]. Interestingly a comparison of findings from two representative household surveys in Afghanistan in 2005 and 2013 revealed that the perceived availability of health care and positive experience with coverage of healthcare needs worsened significantly over the period for people with disabilities [63]. This was despite the availability of a basic package of health services for all.

If the health needs of people with disability are to be prioritized, inclusive health is crucial. Inclusive health encompasses the entire gamut of health care from policies to service delivery [64]. Inclusive health enshrines the principles of efficacy, equity and affordability [64]. The ethos of inclusive health is not just the provision of health services (which may or may not be accessible to people with disability) but affirmative action to ensure that people with disability along with others who are disadvantaged, and discriminated by society receive the due health services so that they can contribute to the overall development of a community. The focus of public health is to respond to the emerging needs of populations including people with disability. Therefore, public health should engage with all stakeholders including people with disability to reduce ill-health, promote optimal health and ensure improved quality of life so that people with disabilities are mainstreamed and not left behind due to their health status.

## 12. Conclusions

South Asia has a significant number of the global people with disability. People with disability in the region report adverse health outcomes and major challenges in accessing health services. These relate both to the health provider prejudices and attitudes, and the inadequacies in skills and infrastructure in caring for people with disabilities. There is an urgent need to find locally-affordable, contextually-specific interventions to improve the quality of health of people with disabilities in the South Asia region.

## Figures and Tables

**Table 1:** Available Disability Data from South Asia.

Country	Disability Prevalence	Year	Data Type	Age	Level	Reference
Afghanistan	2.7%	2015	Survey	All	National	[28]
Bangladesh	9.1%	2015	WG ^a^	All	National	[28]
Bangladesh	10.5	2015	RAD ^b^	All	District	[6]
Bangladesh	4.7%	2009	Survey	All	District	[34]
Bangladesh	8.9%	2010	RAD	18+	District	[35]
Bhutan	3.4%	2015	Census	All	National	[28]
India	2.2%	2015	Census	All	National	[28]
India	12.2%	2015	Survey	All	District Telangana	[11]
India	10.4%	2014	RAD	18+	District South India	[36]
India	6.8%	2014	RAD	18+	District North India	[37]
India	2.9%	2016	RAD	18+	District Assam	[38]
India	9.9%	2016	RAD	18+	Urban Hyderabad	[39]
Maldives	10.9%	2015	WG ^a^	All	National	[28]
Myanmar	2.3%	2015	Census	All	National	[28]
Nepal	1.9%	2015	Census	All	National	[28]
Nepal	5.2%	2001	Survey	All	National	[40]
Pakistan	2.5%	2015	Census	All	National	[28]
Sri Lanka	8.7%	2015	WG ^a^	All	National	[28]
Sri Lanka	3.8%	2014	WG	18+	National	[41]

^a^ Washington Group; ^b^ Rapid Assessment of Disability.

**Table 2:** Health Outcomes in Persons with Disability.

Parameter	People with Disability	No Disability	Remarks	Reference
Nested Case Control study in one district each in Telangana & Karnataka, India using mix of Key Informant Identification and clinical examination				
Ever hospitalized	17.8%	5.0%	*p* < 0.001	[14]
Current medication	9.4%	5.1%	*p* < 0.001	[14]
Known diabetic	12.5%	0.7%	*p* < 0.001	[14]
Feel low constantly	20.7%	2.4%	*p* < 0.001	[14]
Nested Case Control Study in Mahbubnagar district, Telangana, India using mix of clinical diagnosis and self-reported disability. OR: odds ratio; CI: confidence interval.				
Serious health problem in a year’s recall	26%	10%	OR:- 3.2 (95% CI: 2.1–4.8)	[16]
Elevated blood pressure	11%	5%	OR: 1.8 (95% CI: 1.0–3.3)	[16]
Diabetes	5%	3%	OR: 1.5 (95% CI: 0.7–3.3)	[16]
Using Rapid Assessment Tool to identify health needs of people with disability				
Unmet health need (South India)	45.9%	26.8%	*p* < 0.001	[36]
Feel in good health most of the time (South India)	32.9%	41.1%	*p* < 0.001	[36]
Unmet health need (North India)	29.7%	6.4%	OR: 5.5 (95% CI: 2.7 – 11.3)	[37]
Unmet health need (East India)	46.7%	14.5%	*p* < 0.001	[38]
Feel in good health most of the time (South India)	27.8%	60.2%	*p* < 0.001	[45]

**Table 3:** Barriers to accessing healthcare services in South Asia.

Country	Distance	Cost	Poor Communication	Ill Treatment by Providers/Negative Attitudes	Transportation	Inadequate Drugs/Equipment/Buildings	Awareness	Non Availability of Services	Reference
South India (Telangana)				12.6%	13.3%	26.0%	13.3%		[14]
North India	12.1%	10.9%					14.6%		[36]
South India (AP)		40.8%							[6]
East India	16.7%	44.4%		6.7%			7.8%		[38]
